# Tailoring implementation of a youth-focused mental health intervention in Sierra Leone using an implementation blueprint methodology

**DOI:** 10.1186/s12889-024-20896-w

**Published:** 2024-12-18

**Authors:** Alethea Desrosiers, Bidemi Carrol, Jacqueline Hayes, Fatoma Momoh, Haley Ritsema, Hannah E. Frank, Unisa Jalloh

**Affiliations:** 1 Department of Psychiatry and Human Behavior, Brown University, Warren Alport Medical School, 345 Blackstone Blvd, Providence, RI 02906 United States; 2 RTI International, 701 13th St NW #750, Durham, Washington, D.C. 20005 United States; 3 Innovations for Poverty Action, 47A&B Johnson Street, Freetown, Sierra Leone; 4 Save the Children International, St Vincent House, 30 Orange Street, London, WC2H 7HH United Kingdom; 5 Caritas-Freetown, 55 Savage Road, Freetown, Sierra Leone

**Keywords:** Implementation blueprint, Qualitative methods, Schools, LMICs, Adolescents

## Abstract

**Background:**

Identifying contextual factors that might support or hinder implementation of evidence-based mental health interventions for youth in low- and middle- income countries may improve implementation success by increasing the alignment of intervention implementation with local needs and resources. This study engaged community partners in Sierra Leone to: (a) investigate barriers and facilitators to implementing a mental health intervention within Sierra Leone’s schools; (b) develop an implementation blueprint to address identified implementation barriers; (c) explore the feasibility of using the implementation blueprint methodology in Sierra Leone.

**Methods:**

We recruited Ministry of Education Officials (*n* = 2), teachers (*n* = 15) and principals *(*n *=* 15) in Sierra Leone to participate in needs assessment qualitative interviews. We used a rapid qualitative analysis approach to analyze data. Three team members summarized transcripts based on domains aligned with the structured research questions, organized themes into a matrix, and identified and discussed key themes to arrive at consensus. We then reconvened community partners to discuss implementation strategies that could address identified barriers. Participants ranked barriers according to high/low feasibility and high/low importance and selected implementation strategies for the blueprint.

**Results:**

Qualitative results revealed several implementation barriers: teacher/parent/student buy-in; teacher motivation; scheduling time; limited funding; waning interest; daily hardships outside of school. Strategies selected included: develop/distribute educational materials; conduct education meetings/outreach; identify and prepare champions; access new funding.

**Conclusions:**

Engaging community partners to develop an implementation blueprint for integration of a mental health intervention within Sierra Leone’s schools was feasible and may increase implementation effectiveness.

**Supplementary Information:**

The online version contains supplementary material available at 10.1186/s12889-024-20896-w.

Mental health disorders are the second-largest contributor to the global burden of disease among youth and adults [1]. In low-and middle-income countries (LMICs) it is estimated that 90% of mental health disorders remain untreated [2], and in Sub-Saharan Africa, the prevalence of untreated mental health disorders is approximately 85% [3]. While many promising evidence-based mental health interventions have been developed and tested, the implementation process in LMICs is complex. Programs often are not implemented to ensure that they align with locally identified needs and resources and are consequently not effective or sustainable [4]. Identifying alternative delivery settings in which evidence-based mental health interventions can be feasibly delivered and sustained by non-specialists is critical to effectively reduce the incidence of mental health problems. Schools are a viable delivery settings to reach large numbers of youth in LMICs, but more rigorous studies of the implementation context of school settings are needed to better support the adoption and sustainment of evidence-based practices within the education sector of an LMIC.

Using collaborative methods to understand contextual implementation determinants of youth-focused, evidence-based mental health interventions in LMICs may improve implementation success. One such method is to co-create an implementation blueprint with community partners. An implementation blueprint has been defined as a plan that delineates goals, strategies and personnel involved in the implementation process [5]. Key steps of the methodology include: (a) needs assessment of implementation barriers and facilitators, and (b) selecting implementation strategies to address identified barriers and/or leverage facilitators [6, 7]. Prior research in community practice settings in the United States suggests that the implementation blueprint methodology can be feasibly applied with community partners at low-cost, that it can help identify important obstacles to successful implementation of evidence-based interventions in real world settings, and that it can help cultivate a sense of shared ownership in the implementation research process and plans [6–8]. The implementation blueprint can be considered a type of developmental formative evaluation in which determinants to implementation are identified prior to initiating the project. The resultant findings allow researchers to identify potential problems as well as strategies to overcome them prior to launching an implementation effort [30]. These types of developmental formative evaluations that involve community partners during the pre-implementation phase are not often conducted in LMICs, and to our knowledge, the implementation blueprint methodology has not yet been applied in Sub-Saharan Africa.

Additionally, implementation process models, such as the Exploration, Preparation, Implementation Sustainment (EPIS) model [9], can also help structure and guide the collaborative methodology of developing an implementation blueprint to better prepare for the sustainment phase. EPIS is a broad, multi-level, context sensitive implementation process model that has been used successfully to guide implementation of evidence-based interventions in prior research in Sub-Saharan [10, 11], including in Sierra Leone [U19 MH109989]. The implementation blueprint methodology focuses on conducting engagement activities with community partners during the exploration and preparation phases (i.e., pre-implementation), with application of the blueprint during pre-implementation, implementation and sustainment phases. Including key community partners and stakeholders in the implementation planning process has the potential to increase both the ‘sense of trust’ and ‘sense of ownership’ from multiple community groups [12] and thus may increase the likelihood of successful implementation and sustainment of evidence-based mental health interventions for youth within schools in Sierra Leone.

Guided by the methods for developing a tailored implementation blueprint [6, 7], community engagement, and the EPIS model, this study engaged key community partners in Sierra Leone to: (a) conduct a needs assessment of barriers and facilitators to teacher delivery of an evidence-based mental health intervention—the Youth Readiness Intervention (YRI)—within Sierra Leone’s secondary schools; and (b) use modified conjoint analysis to co-create an implementation blueprint to address contextual factors (i.e., implementation determinants) that might influence implementation and sustainment of the YRI within schools in Sierra Leone. We also sought to explore the feasibility of using the implementation blueprint methodology in a resource-constrained, LMIC setting.

## Methods

### Participants and setting

We recruited and enrolled 32 key stakeholder participants in the Western area of Sierra Leone, including government officials (*n* = 2), principals (*n* = 15) and teachers (*n* = 15) from secondary schools in the Western Area during the pre-implementation phase (i.e., prior to YRI implementation). Potential stakeholders were identified in collaboration with local study partners in Sierra Leone working in a non-governmental organization due to their interest and/or investment in the education sector and student well-being in their schools. Trained study research assistants contacted potential participants, explained the purpose of the project, and assessed for eligibility. Inclusion criteria for principals were: aged 18 or older; currently employed as a principal for a secondary school in the Western region of Sierra Leone; willing to attend one qualitative interview session. Inclusion criteria for teachers were: aged 18 or older; currently employed as a teacher at a secondary school enrolled in the study; willingness to attend one qualitative interview session. Inclusion criteria for government officials were: aged 18 or older; currently holding an office within either the Ministry of Basic and Senior Secondary School Education (MBSSE) or the Teachers Service Commission (TSC); willingness to attend one qualitative interview session. Exlcusion critera for all key stakeholders were: not meeting the inclusion criteria. Because this study’s primary focus was on exploring barriers and facilitators to implementation of the YRI at the teacher-level within the context of Sierra Leone’s education sector (e.g., we investigated barriers/facilitators to *teacher-delivery* of the YRI within secondary schools), and not on barriers to adoption of the YRI among youth, we did not include youth participants.

The secondary schools (*N* = 18) where enrolled teachers and principals worked were a mixture of government, private, and faith-based schools, and all were in urban areas. Most were co-educational schools. The two government officials represented the MBSSE and the TSC. All participants provided informed consent prior to study participation. All study procedures were approved by the Brown University Institutional Review Board and the Sierra Leone Ethics and Scientific Review Committee.

### Intervention overview

The YRI is an evidence-based mental health intervention that was culturally developed in Sierra Leone to address the unique mental health needs of the nation’s youth population [13]. It incorporates core components of cognitive behavioral, interpersonal, and mindfulness therapies, and it has demonstrated effectiveness in improving emotion regulation, pro-social functioning, and depression/anxiety symptoms among youth within both school and entrepreneurship training settings [14–16]. The YRI consists of 12, 90-minute sessions that can be delivered 1–3 times per week, depending on the delivery setting, and it can be feasibly delivered by non-specialists with quality.

### Needs assessment procedures

Trained research assistants conducted semi-structured interviews with participants to explore barriers and facilitators to YRI implementation by teachers and sustainability of the YRI within schools. The interview guide explored potential barriers and facilitators across several contextual domains that were drawn from the Framework for Dissemination [17]: (a) resources, (b) norms and attitudes, (c) policies and incentives, (d) and information flow, as well as buy-in and school climate/culture. This framework was selected because it has been used effectively in prior research on the implementation blueprint methodology within a resource-limited community setting [6], and the contextual domains in the framework are relevant in an LMIC school setting. The interview guide was developed for the current study and has not been published elsewhere (see Supplementary Files). Interviews were conducted in-person at their work location and lasted approximately 60 min. Participants received a home gift (equivalent to ~ 18 New Leones) and a stipend for transportation after completing qualitative interviews.

### Needs assessment qualitative data analysis approach

A rapid qualitative analysis approach was used to analyze interview data. This approach is common in implementation research to quickly identify themes for rapid translation and application to a project, particularly when results of qualitative data analysis are needed to guide subsequent phases of a research study [18, 19]. Three coders were involved in the framework-guided qualitative analysis process. To begin, a summary template of the interview guide was created with one section representing each interview question (i.e., YRI fit with school culture, need for YRI, existing and needed resources, relationships and communication among school personnel, training and delivery considerations, sustainability of the YRI). Next, the data from each interview was consolidated using the summary template. To ensure consistency across coders, all coders (BC, JFH, HR) independently coded the same three transcripts initially. Coders then met to clarify coding discrepancies and make final revisions to the summary template to ensure applicability to the interview data. The remaining transcripts were divided evenly between coders to complete independently. Coding meetings were held periodically to address questions and issues as they arose during the coding process. After each interview was consolidated, data were migrated to a framework matrix in which each participant’s data were represented in a row and each interview domain in a column. All three coders then collaborated to identify common themes across participants within each domain. Disagreements were resolved through discussion until coders arrived at general consensus. To inform the modified conjoint analysis (described below), key barriers were distilled into a list based on the identified themes.

### Modified conjoint analysis procedures and analysis

We conducted a modified conjoint analysis in collaboration with these community partners who had participated in the initial needs assessment to discuss the key barriers to implementation and sustainment [6]. We reconvened 12 teachers, two principals, and one TSC official who had participated in the needs assessment to meet for a 90-minute in-person session. Participants received a stipend to offset transportation costs. Modified conjoint analysis is a method in which stakeholders assign values to different aspects of a service or intervention to illuminate “trade-offs” and inform decision making [6, 20]. Two implementation research team members led this process. We invited community partners to join a 90-minute face-to-face meeting in which we described the identified barriers and prioritized barriers by having group members rank them in terms of feasibility (high or low) and importance (high or low). Group members were first provided a list of the identified barriers. We then used a large poster board grid with importance labeled on the Y-axis and feasibility on the X-axis, and placed post-it notes for each barrier in their ranked location on the grid. Rankings were determined through a discussion and consensus process, and then group members identified where on the grid each barrier should be placed. Separate poster boards were used for barriers to implementation and sustainment.

Barriers that were ranked “high importance” and “high feasibility” were prioritized for continued discussion. In advance of the meeting with community partners, the research team developed a ‘menu’ of 38 implementation strategies that might be used to address these barriers. The menu of implementation strategies was pre-selected from the comprehensive ERIC list of 73 implementation strategies [5] by two research team members based on: (1) their relevance to the implementation context of school settings in Sierra Leone; (2) consideration of known resources and resources constraints; and (3) potential to address known barriers. Each implementation strategy was described by the two research team members and then discussed with the group to ensure understanding. Community partner participants identified the strategies from this menu that they thought were the most applicable to address the prioritized barriers (at least one individual endorsement, or one verbal vote of “yes”, was needed to retain the strategy); strategies were then ranked according to feasibility (high or low) and impact (high or low) via a collaborative discussion and consensus process. Once again, post-its for each strategy were placed on the poster board grid according to their ranking. Through further discussion and consensus, implementation strategies were then linked with the barrier that they addressed. Implementation strategies ranked as high feasibility and high impact were noted and included in the implementation blueprint.

### Developing the implementation blueprint

Two members of the research team drafted the implementation blueprint based on the results of the conjoint analysis process and then shared it with stakeholders. Components of the blueprint included: implementation barriers, implementation strategies; action steps, and research team members responsible for carrying out the action steps. Potential action steps for each implementation strategy were discussed with the key stakeholders and other implementation team members (i.e., project coordinator; implementation partner) and included in the implementation blueprint. Although the team identified suggestions for responsible parties for each action step, teachers and principals were also encouraged to identify who would be responsible for taking action steps in their school and whether they could take on specific action steps. Through this same process, we also developed a blueprint for sustainment, with potential action steps for schools to take during the sustainment phase.

The approach for assessing *feasibility* of using the implementation blueprint methodology was via: (a) successful recruitment of the target number of community parnters to participate in the needs assessment interviews; (b) successful reconvening and participation of community partners in the conjoint analysis process; (c) successful development of the blueprints for implementation and sustainment. We defined feasibility based on Proctor’s implementation outcomes framework, where feasibility is the extent to which an implemention method (or target) can be successfully used in a given context [21].

## Results

### Needs assessment participant demographics

Key stakeholders (14 female/18 male) represented 18 schools, and their ages ranged from 23 to 65 years (M = 48, SD = 9.1). Educational backgrounds varied: 20 held undergraduate degrees, 11 had graduate degrees, and one possessed a teaching certificate. We successfully recruited the target number of key stakeholders to participate in the needs assessment, and all potential stakeholders who were invited to participate agreed and consented (*feasibility metric)*.

### Needs assessment results

Participants in the needs assessment interviews indicated that secondary schools in Sierra Leone would benefit from a program like the Youth Readiness Intervention (YRI) to improve interpersonal relationships and coping skills among youth. They identified several facilitators and barriers to the implementation of the YRI, which are described below.

### Barriers

#### Buy-in from teachers, student, and parents

One multi-level barrier mentioned by several needs assessment participants was the potential lack of engagement and buy-in from teachers, students, and parents. They thought that students, particularly those in senior secondary schools, may prioritize academic performance over extracurricular activities. Participants offered ideas about how to address this potential barrier. For example, they suggested that providing clear and transparent communication with parents was identified as critical for program success, potentially increasing student willingness to participate. One participant (principal, male, age 52) said, “If you want to get the involvement of parents, it will be a great idea”. Similarly, another stated, “Sending a letter to [the parents] will help instilling confidence in the process, and I believe they will be ready to send their kids to be part of the program (principal, male, age 50).

#### Teacher motivation and burden

Needs assessment participants stated that igniting initial motivation among teachers to participate in YRI training and deliver YRI sessions, as well as maintaining motivation and interest in providing YRI sessions over time, could both pose challenges to YRI implementation. Teachers, often under-compensated and overworked, could perceive the YRI as an additional burden without clear benefits to themselves or their students. For example, one senior teacher (female, age 46) stated, “Teachers…would need to be encouraged, especially by providing [them] stipends”. Similarly, a principal (acting principal, female, age 48) stated.

*Yeah*,* there are challenges*,* as I have stated*,* among the challenges is…the lack of motivation on the part of the facilitators that is the teachers in the school will lose interest at some point. Sometimes even if you try to force them*,* they will not cooperate especially when it comes to volunteering to take up the program*,* but when it comes to money business they will show interest*,* but volunteering they will not show interest.*

#### Waning interest over time

Participants also discussed their prior experiences with programs like the YRI that were successfully implemented, but not sustained over time. Participants attributed this partly to waning interest among teachers and/or principals after the initial enthusiasm. They also mentioned that difficulties securing ongoing resources to support programs like the YRI resulted in gaps in implementation of programs, and participants thought that interest and enthusiasm often waned during these gaps. One teacher stated.

*At first*,* we embraced them [similar programs] with good spirits though I have not been directly involved in these programs as an individual. These programs will start on a very good note*,* they will accept them*,* and they will just die down quietly. With time you won’t hear anything about them again. There is a huge question mark when it comes to continuity. They are not sustainable*.

#### Time constraints

Participants noted that many schools in the Western region of Sierra Leone have a double-shift system, with junior schools attending in the morning and senior schools in the afternoon. This schedule leaves little time for extracurricular activities, as instructional time is already limited; therefore, stakeholders thought that integrating the 90-minute YRI sessions into the school day could be a major challenge. They also noted challenges with after school programs for schools operating on shifts and students attending the second shift due to safety concerns for students walking home and the limited daylight remaining at the end of the school day. For example, one MBSSE official (male, age 42) stated.

*In double shift systems*,* there you will have the biggest challenge because the day is short and the children would want to go home*,* and the same classrooms will be utilized by the next group*,* so the classrooms are hardly free or hardly empty*,* and when the second session ends it is almost the end of the day.*

Regarding challenges with single shift schools, one teacher (male, 64 years) stated, “A normal school day ends at 3:15 pm, some of us have other things to do, to make some extra finance to run our homes and other affairs like health-wise.”

#### Daily hardships faced by youth

Needs assessment participants noted that food insecurity, unstable home environments, and poverty might impede YRI session participation. For example, one teacher (female, 50 years) noted, “Some of these kids leave their home without any food, so moving them from one place to another or bringing them together, you have to ensure you motivate them by feeding them and providing transportation.” Another teacher (female, 40 years) stated, “We have pupils who are coming from depressed and deprived backgrounds. There are those coming from broken homes. They have lots and lots of problems.” Students from disadvantaged backgrounds may not perceive the immediate benefits of programs like the YRI when faced with more pressing needs, including food insecurity.

#### Limited funding/resources

Participants mentioned that limited funding to support providing programs like the YRI in schools as well as both the financial and human resources to sustain them over time could make it difficult to implement the YRI in schools in Sierra Leone. For example, one teacher (male, 45 years) stated, “One major challenge we do have in this country is resources. If you have the goodwill for a certain program like this to come into effect, but don’t have the resources, that will definitely be a challenge to implement that particular project”. Similarly, a principal (male, 48 years) stated:

*As I said earlier…we…face challenges. One for instance, sometimes is finances. They may want to undertake certain programs/activities, but they do not have the necessary* *funds to facilitate them. When they do come to me*,* I might not have the money they needed*,* nor the teachers that oversee them*,* therefore most times the program will* *either halt or will be suspended for a while until we have the requisite amount of money they need. That is one such challenge.*

### Facilitators

#### Alignment with school mission and values

Participants noted that the YRI’s goals aligned with overall objectives of schools to support student success, foster good citizenship, and improve interpersonal skills. As learning organizations, schools expressed a willingness to embrace new initiatives. One participant said.

*I see the YRI program as a complimentary tool*,* which when properly implemented*,* has the tendency to shape and mold our students into responsible adults as they leave the school and are ready to be integrated into the work class. If we actually give you the attention that is necessary to adopt this*,* then it will be good for our youths’ future developmental plans because it… prepares them for adulthood in their respective areas in which they want to pursue.* (teacher, male, 45 years)

Another participant (teacher, female, 23 years) talked about the potential fit of the YRI content within their school’s culture. “In terms of our culture, our pupils like to participate in such programs…and we used to have such programs…we discuss[ed] with them some of these issues so that we can guide and encourage them.” Likewise, one principal (male, 46 years) talked about the openness in their school to new ideas and innovation, “It is a new initiative, and in our school, we are very much innovative. I think [this] will help make it work well for us”.

#### Experience with similar programs

Schools cited successful experiences with implementing comparable programs, such as mental health clubs, gender-based violence prevention programs, and anti-violence initiatives. This existing experience was perceived as beneficial for YRI implementation. For example, one stakeholder said, “these programs were really successful. Since they had to do with students’ development, the staff in the school embrace[d] them very well and whole-heartedly (principal, male, 50 years).” Another said, “We have anti-violence programs implemented here. Those programs… have their success stories and their challenges as well, but they have been successful (teacher, male, 48 years).”

### Modified Conjoint Analysis results

We successfully reconvened key stakeholders (*N* = 12) to participate in the conjoint analysis process (*feasibility metric)*. Results of the modified conjoint analysis, including ranking of barriers, are presented in Tables 1 and 2. Stakeholders ranked five barriers to implementation as high importance and high feasibility. During the ranking process, stakeholders agreed that some barriers belonged in the middle of the axis for either importance or feasibility, so these barriers were ranked as “medium/moderate” instead of high or low. Three barriers to sustainment were ranked as high importance and high feasibility. For each barrier, stakeholders identified 1–3 implementation strategies that were highly feasible and had the potential for high impact. Ten strategies targeting implementation barriers and seven strategies targeting sustainment barriers were ranked as ‘high/high’. The research team also added several implementation strategies that were pre-planned during development of the research study protocol to the implementation blueprint. These included: conducting outreach with teachers (i.e., meeting with teachers to explain the YRI components and invite them to participate in the study), training teachers in the YRI, providing booster sessions to any teachers who did not demonstrate < 70% competency after training, providing financial incentives to teachers for YRI session delivery, and accessing funding (i.e., implementation activities supported by the grant). The pre-planned implementation strategies were discussed with community partners but were not subject to the ranking process because they were required in the study protocol. Of note, community partners expressed high enthusiasm for the strategy to support sustainment related to identifying and preparing a champion, both at the level of teacher and student, but recognized that they likely needed further support, resources and technical guidance in the future to execute this strategy. The final results of this process were successfully incorporated into the co-created blueprints for implementation and sustainment (*feasibility metric).*

**Table 1:** Blueprint for YRI implementation in schools with action steps

Implementation Barriers	Feasibility	Importance	Implementation Strategy	Action Step (Responsible Party)
Teacher buy-in	H	H	Conduct Educational Meetings	Meetings with teachers to explain the project, the YRI components and prior findings; and recruit interested teachers (Data collection team)
Parent buy-in	H	H	Conduct Educational Meetings; Conduct Outreach Visits	Contacted parents and scheduled home visits to explain the project and the YRI (Data collection team)
Student buy-in/competing academic priorities	M	H	Involve leadership in schools	Sessions scheduled at times to minimize interference with exam schedules & holidays (Principals)
Teacher Motivation	M	H	Alter Incentives; Conduct Ongoing Training; Provide supervision	Teachers provided a mobile money payment after completing all YRI sessions (Implementation partner); Provided two weeks of YRI training and booster sessions; Weekly supervision (Implementation partner)
Scheduling/ Time constraints	H	H	Involve leadership in schools; Tailor strategies	Teachers and Principals in each school identified best time for YRI sessions in their school (Teachers/Principals)
Limited Funding/ Resources	H	H	Access new Funding	Funding used from NIMH grant to support YRI delivery by teachers and supervision of teachers by YRI experts (Academic researchers)
Waning Interest over Time	H	H	Identify and Prepare Champions	Local project coordinator/YRI trainer; visited schools and rallied enthusiasm (Implementation partner)

*Note: ‘*Daily hardships outside of school’ were not included in the blueprint because this identified implementation barrier was ranked as low feasibility

**Table 2:** Initial blueprint for sustainment with potential action steps

Sustainment Barriers	Feasibility	Importance	Sustainment Strategy	Action Step (Responsible Party)
Teacher Motivation	L/M	H	Create a Learning Collaborative; Identify and Prepare teacher Champions; Create educational resources; Identify incentives; Provide ongoing consultation	Review feedback from supervisors on teacher competency to identify teacher champions; create a “cheat sheet” of session reminders (Local project coordinator/YRI trainer); discuss non-financial incentives with teachers & principals (Implementation partner)
Limited Funding/Resources	H	H	Access new Funding; Conduct Educational Meetings; Create educational resources	Conduct meetings with the MBSSE to discuss financing (Teachers & principals); Conduct meetings with school leadership to review implementation costs (Implementation partner)
Waning Interest over Time (youth)	H	H	Identify and Prepare youth champions; Prepare teacher champions	Ask teachers to nominate youth champions from their groups (Teachers & principals); develop resources for champions to use to talk about the YRI in their schools, radio or newspapers (Local project coordinator/YRI trainer)

Note. NIMH-funding used to help identify other sources of funding to support sustainment

## Discussion

To the best of our knowledge, this is the first study to apply the implementation blueprint development methodology in an LMIC setting and to identify determinants of YRI implementation. Findings suggest that the steps of the implementation blueprint creation process (e.g., needs assessment, conjoint analysis, blueprint) were feasible to complete in a low-cost way (i.e., use of posterboard and pens rather than software and computers) with stakeholders in the education sector in Sierra Leone. We successfully engaged stakeholders during the pre-implementation phase, operationalized implementation strategies that targeted identified implementation barriers, conducted conjoint analysis in partnership with key stakeholders, and developed an implementation blueprint that teachers and principals could use to better plan for effective YRI implementation and sustainment within their school.

The initial needs assessment revealed several potential barriers to YRI implementation that were critical to address, particularly time constraints and buy-in from teachers, parents, and youth. Findings are in line with prior studies in high-income countries reporting that constraints on teachers’ time and securing buy-in from multiple stakeholders are critical barriers to implementing evidence-based practices in schools [22–24]. In the current study, implementation strategies that were identified for inclusion in the blueprint to address these barriers included: (a) tailoring implementation strategies (i.e., teachers and principals of each school identified a day/time for YRI sessions in their school) and involving leadership to address time constraints; and (b) educational implementation strategies, including educating parents, teachers and youth and conducting outreach to address buy-in. As per the blueprint, these strategies (action steps) were implemented by the responsible party (i.e., teachers/principals, implementation partners, data collection team), target recruitment numbers for teachers and youth were achieved, and the target number of teachers completed YRI training.

In addition to this evidence suggesting that the process of *developing* a blueprint was feasible, our findings suggest that *carrying out* the blueprint was also feasible, and it may have helped to address some of the identified barriers. Although not specifically evaluated, our perception is that participation in the implementation blueprint creation process facilitated mutual trust, buy-in, and enthusiasm among teachers and principals [25]. In line with the theory of diffusion of innovations [26, 27], these “early stage adopters” may have helped to increase buy-in and generate enthusiasm from others (i.e., teachers, students) in their schools prior to implementation. Future research might explore stakeholder experiences with the implementation blueprint creation process and whether/how this experience shaped their attitudes and behaviors during the implementation process. Interestingly, some stakeholders also offered suggestions for addressing implementation barriers that mapped on to the pre-selected menu of implementation strategies from the ERIC list (i.e., conducting outreach-sending letters home to parents; providing incentives-offer teachers an incentive for session delivery) prior to reviewing the list. The research team noted that these strategies could be especially contextually relevant and feasible, and should perhaps be prioritized when creating the implementation blueprint.

It is also worth noting that, compared to high income countries, additional time and resources were required to engage in modified conjoint analysis activities to ensure that the implementation blueprint methodology was accessible and relevant for the local cultural context and key stakeholders in Sierra Leone. Because the implementation strategies from the ERIC list have been codified based on implementation research that is almost exclusively from high income settings; many strategies listed were not relevant or feasible to carry out in Sierra Leone. Additionally, all of our stakeholders were unfamiliar with implementation science research methods and found that the terminology and wording of many implementations strategies was difficult to understand. The research team spent additional time to define and operationalize the menu of pre-selected strategies in simpler terms and then provided examples relevant to Sierra Leone to improve comprehension. Future research might consider developing and/or operationalizing more LMIC specific implementation strategy lists or engaging local stakeholders in the process of defining implementation strategies. Research focused more on implementation within the health system or health sector in LMICs, rather than the education sector, might refer to the strategies targeting the categories identified in a prior review of implementation strategies for health systems in LMICs [28].

### Limitations

Along with the strengths of this study, there are also limitations to note. Unlike previous studies [6], we did not collect quantitative data on implementation determinants (i.e., organizational climate or culture, attitudes towards evidence-based practices) during the needs assessment phase. Quantitative data collection on readiness to change and organizational climate was planned for the baseline data collection point, which occurred after the implementation blueprint creation process. Devoting more time during the Exploration and Preparation phases to plan what types of implementation determinants or outcomes are most important to assess during each stage of the implementation research process and how they will be assessed could better support future efforts to build implementation blueprints. Additionally, as recommended in prior research [8, 29] planning and using methods to evaluate whether implementation strategies from the blueprint are used in a particular setting as well as whether they are associated with implementation-effectiveness and sustainment is needed to better understand whether the blueprint was effective and why [30]. While we documented that each action step in the implementation blueprint was indeed taken by the respective responsible party, we did not establish a plan to measure whether each strategy related to implementation effectiveness. Our ability to carry out the implementation blueprint methodology with stakeholders in Sierra Leone’s education sector could suggest that these methods are feasible to apply in other similar settings, but findings may not generalize to the education sector in other LMICs. Similarly, because we did not have equal stakeholder representation from all schools enrolled in the study, the variation in organizational climate or attitudes among teachers and/or students across schools may not have been fully captured in the implementation blueprint. Because our recruitment method focused on schools in the Western region, findings may not generalize to other school settings in Sierra Leone, particularly in the rural regions where schools have fewer financial and human resources. We also did not include youth/student stakeholder participants; thus, we may have missed an opportunity to identify additional barriers, facilitators, or strategies to incorporate within the blueprint that were relevant to youth. Additionally, future efforts to apply this methodology in an LMIC might benefit from devoting more attention to strategies that leverage implementation facilitators, rather than focus solely on strategies to target barriers. Our focus on high feasibility/high importance barriers was chosen due to resource constraints; however, this means that there are several barriers and facilitators that were not explicitly addressed with the blueprint and that may need to be revisited as implementation and sustainment efforts progress. Finally, we acknowledge that NIMH grant funding is not a long-term solution to supporting the sustainment of the intervention; however, it is an important intermediate step to assess the extent to which we can support sustainable delivery of the YRI with minimal resources (i.e., small incentives for teachers and snacks for students) and to identify how schools might obtain these limited resources. In LMICs, initial start-up funds are often needed from external donors to support initial evidence-based program implementation and evaluation, as well as to support collection of cost data. Future work will also focus on identifying how the intervention can be sustained without the roles that were delegated to researchers in the current project.

## Conclusion

Considering that there are often greater barriers to implementation and sustainment of evidence-based mental health interventions in LMICs and other resource-constrained settings compared with high income settings, testing methodologies to increase stakeholder engagement during the pre-implementation phase to better identifying and address potential barriers to both implementing and sustaining interventions within specific contexts is critical. Future studies might also consider adapting and tailoring tools that are simple and easy to use to evaluate the effectiveness of implementation strategies so that community partners can better consolidate resources towards effect strategies that contribute to sustainment of evidence-based practices.

## Electronic supplementary material

Below is the link to the electronic supplementary material.

Supplementary Material 1

## Data Availability

Data are available upon reasonable request to the corresponding author.

## Abbreviations

LMIC: Low- and middle-income country
EPIS: Exploration, preparation, implementation, sustainment
YRI: Youth Readiness Intervention
MBSSE: Ministry of Basic and Senior Secondary Education
