# Supplementary material for: Tailoring implementation of a youth-focused mental health intervention in Sierra Leone using an implementation blueprint methodology
Source: BMC Public Health. 2024 Dec 18;24:3418. doi: 10.1186/s12889-024-20896-w (PMC11653831; doi:10.1186/s12889-024-20896-w)
Supplement: Supplementary file 1 — Supplementary Material 1 [file 12889_2024_20896_MOESM1_ESM.docx]

# Semi-Structured Interview Guide: Needs Assessment

WELCOME/PREAMBLE

Thank you again for agreeing to share your thoughts about implementing the evidence-based Youth Readiness Intervention for youth within Sierra Leone’s schools. In order to help me understand what things might facilitate implementation and what things might make it hard, I would like to ask you some questions.

In order to remember what we talk about, I will audio-record our conversation and I will write notes. I will then write a report of what was said in our interview. Your name will not be included on the report, and I will not tell anyone what you said today. When the results are reported, all responses will be de-identified. Do you have any questions?

Remember, this is your time and we want to hear from you. There are no right or wrong answers. Please feel comfortable to talk about your thoughts, opinions, and experiences openly.

By sharing your ideas today, we hope to better understand how we can help schools to provide programs to improve the ability of youth to cope with challenges in health ways and manage their emotions. We also hope to learn more about how to address barriers to successfully implementation. Do you have any questions?

Now let’s get started.

# How would you describe the culture (e.g., values, beliefs, assumptions) in your school?

- - How do you think your school’s culture might affect delivery of the Youth Readiness Intervention? What might help make it work well? What might make it hard?
  - To what extent are new ideas or programs embraced and used at your school? To what extent have new programs helped improve your school?
  - What challenges, if any, did you face in being a facilitator?

# In what ways, if any, is there a need for programs like the Youth Readiness Intervention in your school?

- - How do teachers and staff fell about any current programs that are available in your school to help youth cope with challenges they face?
  - In what ways, if any, could the intervention help meet the needs of teachers in your school?
  - To what extent do you believe the intervention could help you improve their classroom behavior? Their relationships with peers?

# How well does the Youth Readiness Intervention fit with existing values and practices in your school?

- - What things, if anything, were particularly good or helpful about the intervention for the kinds of problems Sierra Leonean youth have?
  - What things, if anything, might help the intervention fit well within the existing practices in your school?
  - What things, if anything, might help make it easier to provide the intervention after school?
  - What things, if anything, might make it hard for the intervention to fit within existing practices in your school? What things
  - What things, if anything, felt like they didn’t get enough attention or that youth needed more time to discuss?

# What kinds of resources do you think your school would need to deliver the Youth Readiness Intervention? (Resources can include money, training, physical space, time)

1. **In what ways, if any, could digital technology like mobile apps help provide feedback to teachers on their performance?**

- What might help make it easier for teachers to use mobile tools to help them in their teaching or classroom management skills?
- What would your school need to help teachers use mobile tools to assist them in delivering the school curriculum or after school programs?
- What might make it hard for teachers to use mobile tools to help them deliver the school curriculum or after school programs?
- How willing do you think teachers in your school would be to learn how to use a mobile app to help them with their teaching? What incentives or resources would teachers need?

# Please tell me about your working relationships with other teachers and staff in your school.

- To what extent do you meet with other teachers and/or staff in your school?
- How do you typically find out new information related to what happens in your school (e.g., new staff hired, new initiatives, staff departures)?

# How willing do you think teachers in your school would be to deliver the Youth Readiness Intervention in your school?

- - What challenges, if any, might teachers facing in participating in training and delivering the intervention after school?
  - Amount of time spent after school, privacy, scheduling, incentives?
  - To what extent do teachers in your school have enough time to learn new things like the Youth Readiness Intervention?
  - To what extent do teachers in your school have enough time to participate in supervision to receive feedback on their performance?

# What else, if anything, might help providing the Youth Readiness Intervention in schools work well? What else, if anything, might make it difficult to provide the intervention in schools successfully?

1. **How interested are you in learning more about the Youth Readiness Intervention?**
   - How do you feel about your school providing the Youth Readiness Intervention?
